# Racial and geographic variation in coronary heart disease mortality trends

**DOI:** 10.1186/1471-2458-12-410

**Published:** 2012-06-06

**Authors:** Richard F Gillum, Alem Mehari, Bryan Curry, Thomas O Obisesan

**Affiliations:** 1 Department of Medicine, Howard University College of Medicine, Washington, DC, USA; 2 Division of Geriatrics, Howard University Hospital, Towers Bldg. 2041 Georgia Ave, NW, Washington, DC 20060, USA

**Keywords:** Coronary heart disease, African Americans, Mortality

## Abstract

**Background:**

Magnitudes, geographic and racial variation in trends in coronary heart disease (CHD) mortality within the US require updating for health services and health disparities research. Therefore the aim of this study is to present data on these trends through 2007.

**Methods:**

Data for CHD were analyzed using the US mortality files for 1999–2007 obtained from the US Centers for Disease Control and Prevention. Age-adjusted annual death rates were computed for non-Hispanic African Americans (AA) and European Americans (EA) aged 35–84 years. The direct method was used to standardize rates by age, using the 2000 US standard population. Joinpoint regression models were used to evaluate trends, expressed as annual percent change (APC).

**Results:**

For both AA men and women the magnitude in CHD mortality is higher compared to EA men and women, respectively. Between 1999 and 2007 the rate declined both in AA and in EA of both sexes in every geographic division; however, relative declines varied. For example, among men, relative average annual declines ranged from 3.2% to 4.7% in AA and from 4.4% to 5.5% in EA among geographic divisions. In women, rates declined more in later years of the decade and in women over 54 years. In 2007, age-adjusted death rate per 100,000 for CHD ranged from 93 in EA women in New England to 345 in AA men in the East North Central division. In EA, areas near the Ohio and lower Mississippi Rivers had above average rates. Disparities in trends by urbanization level were also found. For AA in the East North Central division, the APC was similar in large central metro (−4.2), large fringe metro (−4.3), medium metro urbanization strata (−4.4), and small metro (−3.9). APC was somewhat higher in the micropolitan/non-metro (−5.3), and especially the non-core/non-metro (−6.5). For EA in the East South Central division, the APC was higher in large central metro (−5.3), large fringe metro (−4.3) and medium metro urbanization strata (−5.1) than in small metro (−3.8), micropolitan/non-metro (−4.0), and non-core/non-metro (−3.3) urbanization strata.

**Conclusions:**

Between 1999 and 2007, the level and rate of decline in CHD mortality displayed persistent disparities. Declines were greater in EA than AA racial groups. Rates were greater in the Ohio and Mississippi River than other geographic regions.

## Background

In 2009, despite a long-term decline in death rates, coronary heart disease (CHD) was the leading cause of death in the US [1-4]. Although reductions in gender disparities in CHD mortality have been noted [5], disparities in CHD mortality based on race/ethnicity have remained largely unchanged [6], and disparities in the morbidity of CHD appear to be increasing [7]. Many publications have examined the decline in CHD mortality in the US prior to 2000, but relatively few have detailed both racial and geographic variation [1,8-20]. The Healthy People 2010 Initiative set the goals of reducing the CHD mortality rate by 20 percent between 2000 and 2010 and reducing disparities in CHD mortality. The *Healthy People 2010* objectives of reducing death rates to 162 deaths per 100,000 populations for CHD were met in 2004. However, despite the overall decrease in CHD death rates, the target death rates were not met for two subpopulations: blacks and men [3]. Two out of the four overarching goals of *Healthy People 2020* are: 1) achieve health equity, eliminate disparities, and improve the health of all groups; and; 2) Examining and monitoring the distribution of death rates provides the requisite information for focusing on the groups most in need of early intervention to eliminate preventable disease, disability, and premature death and to improve the health of all groups [4]. Magnitudes, geographic and racial variation in trends in CHD mortality within the US require frequent updating for health services and health disparities research.

Accordingly, this report presents CHD mortality trends between 1999–2007 among non-Hispanic blacks and whites aged 35–84 years and geographic and racial variation in CHD mortality trends.

## Materials and methods

Deaths in 1999–2007 with coronary heart disease (International Classification of Disease 10^th^ revision [ICD-10] codes I20-I25 (1999–2007) as underlying cause were enumerated [20]. Data available for public use from National Vital Statistics System (NVSS), files provided the underlying cause of death and demographic data on decedents [20]. The underlying cause of death is the disease (or injury) that initiated the sequence of events leading directly to death. For non-Hispanic African Americans (AA) and European Americans (EA), age-adjusted death rates per 100,000 using the 2000 US standard population with 95% confidence intervals (CI) were computed for persons aged 35–84 years using standard methods [20]. Following policy of the NVSS, years and areas were combined to avoid producing death rates considered unreliable, i.e. when the death count is less than 20. Variation by sex and race in annual percentage change (APC) between 1999 and 2007 were assessed using JoinPoint software [21]. Joinpoint is statistical software for the analysis of trends using joinpoint models, that is, models where several different lines are connected together at the "joinpoints". This enables the user to test that an apparent change in trend is statistically significant. The tests of significance use a Monte Carlo Permutation method. Numbers of U.S. residents for the period 1999–2007 were obtained from the U.S. Bureau of the Census and used to calculate death rates per 100,000 populations. Bridged-race Census estimates were used as denominators for groups defined by race and Hispanic origin. Urbanization levels and Census divisions are defined in Additional file 1: Table S1 [22].

## Results

In 2007 at ages 35–84, US age-adjusted CHD death rates per 100,000 (95% CI) were as follows: women EA 111 (110–112), AA 171 (168–174); men EA 238 (237–239), AA 312 (307–316). Examination of annual percent change (APC) and US age-adjusted CHD death rates between 1999 and 2007 for women and men at ages 35–84 yrs revealed that rates declined for AA and EA men at ages 35–84, and in each age subgroup (Table 1). In both EA and AA women, the rates declined more rapidly in later years compared to the earlier years of the decade. In women, APC was greater above age 54 than at 35–54 years, which the APC for EA women barely different from 0 (Table 1).

**Table 1:** Annual percent change age-adjusted rates of mortality from coronary heart disease per 100,000 by race in non-Hispanic women and men aged 35-84+ years: United States, 1999-2007

**Characterstic**	**Year**	**AA women**	**EA women**	**AA men**	**EA men**
APC(CI)35-84Y	1999-2007	-	-	−4.0 (−4.6,-3.4)	−4.8 (−5.1,-4.5)
Rate	1999	-	-	329	346
Rate	2007	-	-	246	240
APC(CI)35-84Y	2003-2007	-	−6.2 (−7.2, -5.1)	-	-
Rate	2003	-	143	-	-
Rate	2007	-	111	-	-
APC(CI)35-84Y	1999-2003	-	−4.5 (−5.5,-3.5)	-	-
Rate	1999	-	172	-	-
Rate	2003	-	143	-	-
APC(CI)35-84Y	2002-2007	−6.3 (−7.0,-5.6)	-	-	-
Rate	2002	237	-	-	-
Rate	2007	171	-	-	-
APC(CI)35-84Y	1999-2002	−3.7 (−5.2, -2.1)	-	-	-
Rate	1999	265	-	-	-
Rate	2002	237	-	-	-
APC(CI) 35-54Y	1999-2007	−3.2 (−3.9,-2.6)	−0.8 (−1.4, -0.1)	−2.6 (−3.2.-2.0)	−2.1 (−2.4,-1.7)
APC(CI) 55-84Y	1999-2007	−5.7 (−6.3,-5.1)	−5.7 (−6.2, -5.2)	−4.3 (−4.9,-3.6)	−5.2 (−5.5,-4.9)
APC(CI) >84Y	1999-2007	−5.2 (−6.2,-4.2)	−5.9 (−6.5,-5.2)	−6.1 (−6.8,-5.5)	−5.2 (−5.7,-4.8)

AA, African American; EA, European American; APC, annual percent change; CI, 95% confidence interval; Y = ages in years.

Table 2 shows states with in order of decreasing age adjusted CHD mortality rates for AA in 2005–2007, while Additional file 2: Tables S2 and Additional file 3: Table S3 show state rankings for EA. Relative variation was greatest in AA men and least in AA women. For the entire period 1999–2007, Figures 1 and 2 show that high death rates for EA men and women tended to cluster around the Ohio and lower Mississippi Rivers and low rates in Rocky Mountains ( Additional file 2: Tables S2 and Additional file 3: Table S3). Clustering of high rates for both AA men and women was seen in the East North Central, West South Central states, Middle Atlantic States and California and low rates in South Atlantic and East South Central states (see Additional file 4: Figures S1 and Additional file 5: Figure S2. The ratio of the 90th to the 10th percentile of rates in states for EA and AA combined was 1.67 in 1999 and 1.76 in 2007 (1.61 and 1.67 resp. in EA only).

**Table 2:** States* with the highest and lowest age-adjusted coronary heart disease mortality rate per 100,000 by sex and race in non-Hispanic African Americans aged 35–84 years: United States, 2005-2007

	**Women**	**Men**
	Michigan	Michigan
	New York	Oklahoma
	Arkansas	Arkansas
	Oklahoma	California
	California	Tennessee
	Tennessee	New York
	Missouri	Missouri
	Illinois	Pennsylvania
	Ohio	Illinois
	Texas	Mississippi
	Maryland	Louisiana
	Mississippi	Indiana
	Louisiana	Texas
	Kentucky	Ohio
	Arizona	West Virginia
	Pennsylvania	Kentucky
	New Jersey	Maryland
	Florida	North Carolina
	Indiana	New Jersey
	Delaware	South Carolina
	North Carolina	Delaware
	Kansas	Florida
	South Carolina	Arizona
	Virginia	Wisconsin
	Nevada	Virginia
	Alabama	Kansas
	Washington	Washington
	Georgia	Alabama
	Connecticut	Connecticut
	Colorado	Nevada
	Massachusetts	Georgia
	Connecticut	Massachusetts
		Colorado
		Minnesota
Ratio^+^	2.1	3.1

*Excludes states with 99 or fewer deaths in 2005–2007 for given group.

^+^Ratio of rate of state in first row to state in last row.

**Figure 1:**
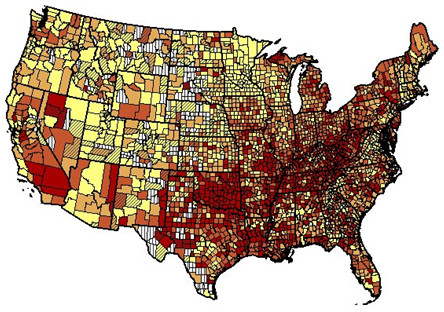
**Age-adjusted rate per 100,000 resident population of death from coronary heart disease by county for European American women aged 35–84 years: United States, 1999–2007.** Yellow, 34.7-114.5; light orange 114.6-142.0; dark orange 142.1-172.2; red 172.4-407.2; white vertical bars, suppressed due to small number of deaths; color diagonal bars, unreliable value.

**Figure 2:**
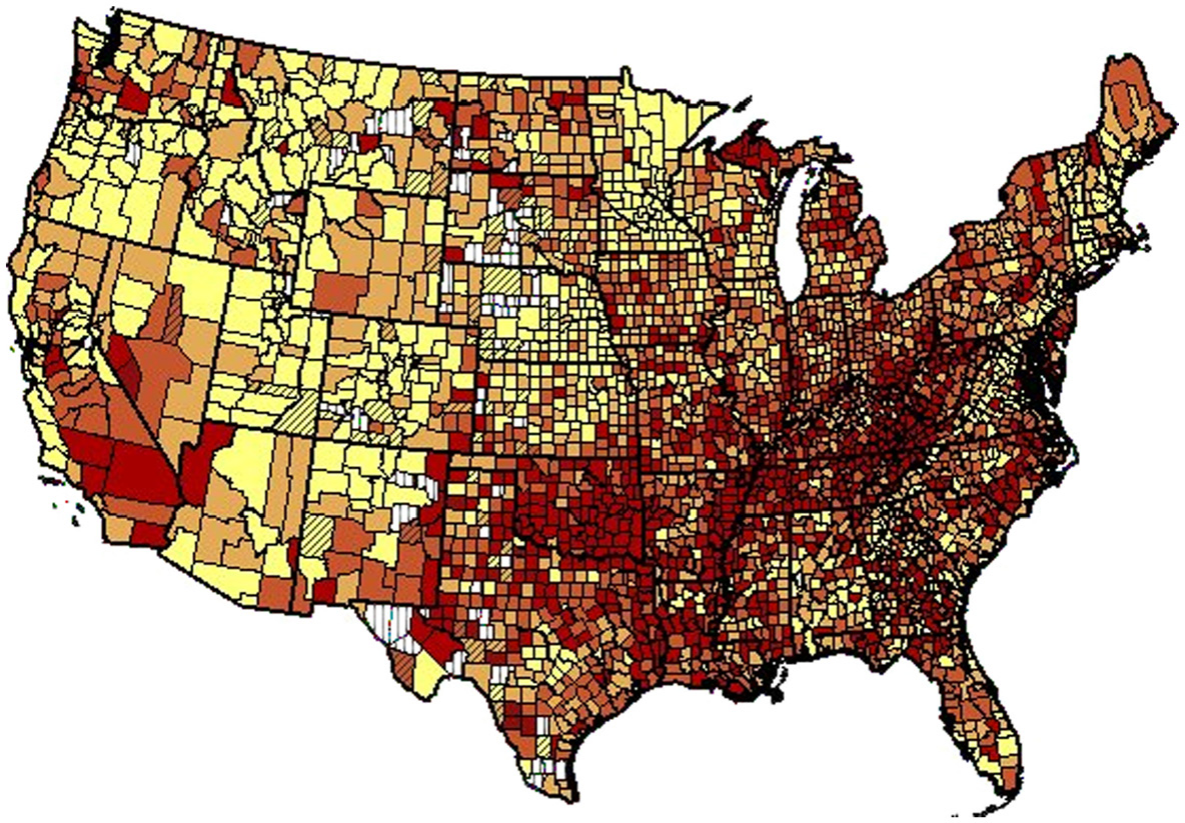
**Age-adjusted rate of death per 100,000 population from coronary heart disease by county for European American men aged 35–84 years: United States, 1999–2007.** Yellow 56.8-252.0; light orange 252.1-300.4; dark orange 300.6-356.3; red 356.4-743.4; white vertical bars, suppressed due to small number of deaths; color diagonal bars, unreliable value.

In 1999–2007 rates declined in all the nine US Census divisions (Table 3). Relative declines tended to be greatest in the New England, Mountain and Pacific divisions. Variation in rate of decline was greater in AA than in EA. The greatest relative declines were in AA women in the East South Central and New England divisions and the smallest relative declines occurred in AA males in the Pacific, Mid-Atlantic and North Central divisions.

**Table 3:** Annual percent change and selected age-adjusted rates of mortality from coronary heart disease per 100,000 by Census division, sex and race in non-Hispanics aged 35–84 years: United States, 1999-2007

**Division**		**AA Women**	**EA Women**	**AA Men**	**EA Men**
New England					
APC	1999-2007	−6.2	−5.9	−4.7	−5.5
Rate	1999	176	144	329	306
Rate	2007	111	93	230	202
Mid Atlantic					
APC	1999-2007	−3.9	−5.4	−3.5	−4.9
Rate	1999	284	197	452	379
Rate	2007	203	124	345	259
E. N. Central					
APC	1999-2007	−5.2	−5.6	−3.6	−5.0
Rate	1999	285	181	487	374
Rate	2007	187	113	352	248
W. N. Central					
APC	1999-2007	−4.9	−5.3	−3.5	−4.8
Rate	1999	274	151	428	324
Rate	2007	175	95	319	217
S. Atlantic	AAPC				
APC	1999-2007	−6.1	−5.8	−4.7	−5.1
Rate	1999	238	165	395	344
Rate	2007	148	102	273	227
E. S. Central					
APC	1999-2007	−6.4	−4.5	−4.1	−4.4
Rate	1999	266	189	434	398
Rate	2007	162	132	309	276
W. S. Central					
APC	1999-2007	−5.8	−4.6	−4.1	−4.5
Rate	1999	276	189	461	385
Rate	2007	172	130	332	267
Mountain					
APC	1999-2007	−5.7	−4.8	−5.6	−4.6
Rate	1999	229	137	332	295
Rate	2007	140	89	193	196
Pacific					
APC	1999-2007	−5.3	−5.5	−3.2	−4.8
Rate	1999	308	172	471	339
Rate	2007	195	107	346	224

AA, African American; EA, European American.

For AA within the Mid-Atlantic, East North Central and Pacific divisions where over 6 million AA aged 35–84 resided, age-adjusted rates for 1999–2007 combined were highest in large, central metropolitan (LCM) areas, e.g. Mid-Atlantic LCM 439 (95% CI 436–443) versus large fringe metro (LFM) 372 (366–379). In South Atlantic and East South Central divisions, rates showed a similar pattern but at somewhat lower levels e.g. South Atlantic LCM 369 (365–373) versus LFM 311 (307–315). In New England, West North Central and West South Central division’s rates were highest in the non-core (non-metropolitan) areas.

For EA within the New England, Mid-Atlantic, East North Central and Pacific divisions, age-adjusted rates for 1999–2007 combined were highest in LCM areas, e.g. Mid-Atlantic LCM 276 (95% CI 274–278) versus LFM 209 (208–210). In West North Central, South Atlantic, East South Central and West South Central divisions rates were highest in the non-core(non-metropolitan) divisions, e.g. East South Central non-core(non-metropolitan) division 281 (278–284) versus LCM 212 (209–215).

Trends were examined by urbanization level in the East North Central division for AA and the East South Central division for EA, i.e. the divisions with highest rates. For AA in the East North Central division, the APC was similar in large central metro (−4.2), large fringe metro (−4.3), medium metro urbanization strata (−4.4), and small metro (−3.9). The somewhat higher APC in the micropolitan/non-metro (−5.3), and especially the non-core/non-metro (−6.5) urbanization strata must be viewed with caution due to much smaller annual numbers of deaths.

For EA in the East South Central division, the APC was higher in large central metro (−5.3), large fringe metro (−4.3) and medium metro urbanization strata (−5.1) than in small metro (−3.8), micropolitan/non-metro (−4.0), and non-core/non-metro (−3.3) urbanization strata.

## Discussion

The long-term decline in CHD mortality rates has continued during the first decade of the new century for major demographic subgroups. However, the historic racial disparities and substantial geographic disparities persist. Hence, the Healthy People 2010 goal of a 20% decline between 2000 and 2010 likely will be met or exceeded (2000–2007 all ages decline 33%), but little or no progress towards the goal of elimination of racial or geographic relative disparities during the decade is evident.

Many studies have documented the magnitude and the important contributions of both improvements in health care and improvements in population risk factor levels in reducing CHD incidence rates and reducing case fatality rates for acute myocardial infarction and long-term survival with CHD [1,3-5,8,13-18]. Combined US racial and geographic variation in levels and trends in CHD mortality or morbidity has been the subject of a smaller number of studies [1,8-19]. The causes of the large racial and geographic variation in CHD mortality have yet to be fully identified [23-30]. Disparities in access to and quality of therapeutic and preventive health care and socioeconomic and cultural variation in lifestyles and risk factor levels are thought to play important roles [29,30]. For example, persistent racial disparities in utilization of revascularization produces have been well documented [31,32].

Early investigators noted high stroke mortality rates among Americans in the southeastern coastal states. By late 1980s, this “stroke belt” had dissipated in the south eastern coastal areas and shifted to the Midwest regions of the Mississippi and Ohio River valleys [8]. CHD mortality has shown a similar westward shift to the so called coronary valley [9]. The increased cardiovascular disease (CVD) mortality among blacks has been mainly confined to the southern states (Mississippi River valley). Although changes in regional profile of risk factors, local environment, and migration pattern may have played a role, recent economic shifts in these areas may be the principal reason for changes in disease rates [8]. Whereas southeastern coastal areas have undergone considerable economic development, the more westward regions have not kept pace [8]. In Mississippi, one of the most economically and educationally disadvantaged US states, CVD mortality has risen among African Americans over the past two decades while among whites there has been a decline [10]. Similar patterns are also observed in the Northeast and the Midwest [11,12].

Our understanding of the existence in race/ethnic variations in CHD disease burden comes from observed differences in their rates and risk factors between countries. For example, in the Seven Countries Study, [33], low CHD rates were observed in Japan and the Mediterranean countries, and high CHD rates in Finland and the US. These differences were in large part explained by differences in risk factors, but several factors may contribute to these observed differences, such as diverse demographic profiles, environmental factors, and genetic factors differences. These variations are perhaps best illustrated by the knowledge gained from studies in migrants. The Ni-Hon-San study of Japanese migrants revealed how blood cholesterol levels and CHD rates rose from relatively low levels among those in Japan, to intermediate levels in Honolulu, and to high levels in San Francisco [34].

Comparison of Afro-Caribbeans, South Asians, and Europeans in the UK indicate marked differences in central obesity, glucose intolerance, hyperinsulinemia, and related dyslipidemia, despite similar blood pressure, body mass index, and total plasma cholesterol [35]. In Canada, there are marked differences between different ethnic groups in the prevalence and death rates from CHD, with the highest rates being among those of European and South Asian origin, but lowest among those of Chinese origin [36], suggesting that the propensity to CHD may vary in different ethnic groups.

Differences in the age-adjusted death rates vary widely between European populations. Data from the World Health Organization (WHO) indicate that the cardiovascular disease mortality rate is 6-fold higher among men and women in the Russian Federation compared with people in France [37]. In 1996, the age-standardized mortality rates for coronary heart disease (CHD) among males in the Russian Federation was 390/100 000 compared with 60/100 000 among males in France [37].

Although the CVD mortality rates are much lower among women compared with men, similar variations among women between countries also exist. Eastern European countries such as the Ukraine, the Russian Federation, Hungary, and the Czech Republic have among the highest and increasing CVD rates in the world, which is in marked contrast to most economically stable European countries where declines in CVD mortality rates have been experienced over the past 30 years [38]. CVD among European populations is mainly attributable to classical risk factors, namely diets high in saturated fats, elevated serum cholesterol and blood pressure (BP), diabetes, and smoking [39].

Strengths of this study include focus on the years 1999–2007 when only ICD-10 was in use, which precluded bias due to changes in ICD version [20]. Unlike some previous studies, analyses were restricted to non-Hispanics to avoid bias arising from misclassification of Hispanic ethnicity on death certificates. However, given the lack of information on racial and regional variation in validity of CHD diagnoses from death certificates, bias due to diagnostic misclassification cannot be excluded as a possible contributor to variation [40]. The validity of using age-adjusted rates for ages 65 yrs and over may be a problem as rates are driven by oldest age groups for CHD where diagnosis accuracy may be questionable. Results of trends in age-adjusted rates within three age bands were performed for this reason. Ecologic analyses of clinical information for cases or data on access to care or risk factor levels, socioeconomic and health service variables in different geographic areas could have confounded or mediated some of the results but analysis of those variables was beyond the scope of this study. More such efforts are needed to explain observed racial and geographic patterns. Continued monitoring of racial and geographic disparities will be of especial importance in an era of health care system changes.

## Conclusions

Between 1999 and 2007, the level and rate of decline in CHD mortality displayed persistent disparities. Rates were greater in the Ohio and Mississippi River than other geographic regions. Declines were greater in EA than AA racial groups.

## Competing interests

The authors declare that they have no competing interests.

## Authors' contributions

RG has been involved in conception of the study, analysis and interpretation of data and drafting the manuscript and revising it critically for important intellectual content. AM and TO have participated in analysis, interpretation of data and revising the manuscript critically for important intellectual content. BC participated in reviewing the content of the manuscript. All authors read and approved the final manuscript.

## Funding

Dr. Obisesan was supported by career development award # AG00980, research award #RO1-AG031517 from the National Institute on Aging and research award #1UL1RR03197501 from the National Center for Research Resources.

## Pre-publication history

The pre-publication history for this paper can be accessed here:

http://www.biomedcentral.com/1471-2458/12/410/prepub

## Supplementary Material

Additional file 1 **Table S1.** Classification rules used to assign counties to the six urbanization levels of the 2006 NCHS Urban-Rural Classification. Click here for file

Additional file 2 **Table S2.** Age-adjusted coronary heart disease mortality rate per 100,000 by state in non-Hispanic European American women aged 35-84 years: United States, 2005-2007. Click here for file

Additional file 3 **Table S3.** Age-adjusted coronary heart disease mortality rate per 100,000 by state in non-Hispanic European American men aged 35-84 years: United States, 2005-2007. Click here for file

Additional file 4 **Figure S1.** Age-adjusted rate per 100,000 resident population of death from coronary heart disease by state for African American women aged 35–84 years: United States, 2005–2007. Legend: yellow 63-139, light orange >141-170, dark orange >173-188, red >199-259. Click here for file

Additional file 5 **Figure S2.** Age-adjusted rate of death per 100,000 population from coronary heart disease by state for African American men aged 35–84 years: United States, 2005–2007. Legend: yellow 88–238, light orange >243-304, dark orange >320-356, red >360-583. Click here for file
