# Supplementary material for: Racial and geographic variation in coronary heart disease mortality trends
Source: BMC Public Health. 2012 Jun 6;12:410. doi: 10.1186/1471-2458-12-410 (PMC3532343; doi:10.1186/1471-2458-12-410)

SUPPLEMENTAL MATERIAL

| **Table S1. Classification rules used to assign counties to the six urbanization levels of the 2006 NCHS Urban-Rural Classification** | |
| --- | --- |
| Urban-rural category | Classification rules |
| **Metropolitan** |  |
| Large central metro^1^ | Counties in a metropolitan statistical area of 1 million or more population:  1) that contain the entire population of the largest principal city of the metropolitan statistical area, or  2) whose entire population resides in the largest principal city of the metropolitan statistical area, or  3) that contain at least 250,000 of the population of any principal city in the metropolitan statistical area |
| Large fringe metro | Counties in a metropolitan statistical area of 1 million or more population that do not qualify as large central |
| Medium metro | Counties in a metropolitan statistical area of 250,000 to 999,999 population |
| Small metro | Counties in a metropolitan statistical area of 50,000 to 249,999 population |
| **Nonmetropolitan** |  |
| Micropolitan | Counties in a micropolitan statistical area |
| Noncore | Counties that are neither metropolitan nor micropolitan |

^1^There must be at least one large central county in each large metro area.

Table S2. Age-adjusted coronary heart disease mortality rate per 100,000 by state in non-Hispanic European American women aged 35-84 years: United States, 2005-2007

| State | State Code | Deaths | Population | Crude Rate | Age Adjusted Rate | Age Adjusted Rate Lower 95% Confidence Interval | Age Adjusted Rate Upper 95% Confidence Interval |
| --- | --- | --- | --- | --- | --- | --- | --- |
| Oklahoma | 40 | 4636 | 2227652 | 208 | 174 | 169 | 179 |
| Tennessee | 47 | 7170 | 4045305 | 177 | 158 | 154 | 162 |
| West Virginia | 54 | 2797 | 1479554 | 189 | 158 | 152 | 163 |
| New York | 36 | 19882 | 10400153 | 191 | 153 | 151 | 155 |
| Arkansas | 5 | 3282 | 1826844 | 180 | 150 | 144 | 155 |
| Kentucky | 21 | 4946 | 3097122 | 160 | 146 | 142 | 150 |
| Mississippi | 28 | 2459 | 1454669 | 169 | 142 | 136 | 147 |
| Rhode Island | 44 | 1341 | 758994 | 177 | 142 | 134 | 149 |
| Missouri | 29 | 6376 | 4016231 | 159 | 138 | 134 | 141 |
| Michigan | 26 | 10034 | 6607551 | 152 | 135 | 132 | 138 |
| Ohio | 39 | 12617 | 8034413 | 157 | 135 | 132 | 137 |
| Maryland | 24 | 4207 | 2894335 | 145 | 130 | 126 | 134 |
| Texas | 48 | 13823 | 9637353 | 143 | 129 | 127 | 131 |
| Louisiana | 22 | 3325 | 2271297 | 146 | 128 | 123 | 132 |
| Indiana | 18 | 6118 | 4327793 | 141 | 126 | 123 | 129 |
| California | 6 | 21141 | 14084624 | 150 | 125 | 124 | 127 |
| New Jersey | 34 | 7567 | 4960576 | 153 | 123 | 120 | 126 |
| Iowa | 19 | 3351 | 2224728 | 151 | 122 | 118 | 126 |
| Delaware | 10 | 765 | 526440 | 145 | 121 | 112 | 130 |
| Pennsylvania | 42 | 14111 | 9033598 | 156 | 121 | 119 | 123 |
|  |  | 239422 | 171371666 | 140 | 119 | 119 | 120 |
| Alabama | 1 | 3807 | 2733028 | 139 | 118 | 114 | 121 |
| Nevada | 32 | 1613 | 1274017 | 127 | 116 | 111 | 122 |
| Illinois | 17 | 9620 | 7135657 | 135 | 115 | 112 | 117 |
| New Mexico | 35 | 1042 | 777772 | 134 | 110 | 103 | 116 |
| North Carolina | 37 | 6515 | 5246953 | 124 | 110 | 108 | 113 |
| Arizona | 4 | 4592 | 3293390 | 139 | 109 | 106 | 112 |
| Florida | 12 | 16277 | 10387342 | 157 | 109 | 107 | 111 |
| South Carolina | 45 | 3047 | 2462076 | 124 | 107 | 104 | 111 |
| Wyoming | 56 | 405 | 362919 | 112 | 107 | 97 | 118 |
| South Dakota | 46 | 705 | 551973 | 128 | 104 | 96 | 111 |
| Vermont | 50 | 569 | 518138 | 110 | 104 | 96 | 113 |
| District of Columbia | 11 | 125 | 126593 | 99 | 103 | 85 | 122 |
| New Hampshire | 33 | 1082 | 1056316 | 102 | 101 | 95 | 107 |
| Virginia | 51 | 4818 | 4435667 | 109 | 101 | 98 | 104 |
| Washington | 53 | 4393 | 4154917 | 106 | 101 | 98 | 104 |
| Kansas | 20 | 2095 | 1835498 | 114 | 98 | 93 | 102 |
| Georgia | 13 | 4569 | 4607765 | 99 | 96 | 94 | 99 |
| Maine | 23 | 1205 | 1127885 | 107 | 95 | 90 | 101 |
| North Dakota | 38 | 545 | 455402 | 120 | 95 | 87 | 103 |
| Idaho | 16 | 945 | 964123 | 98 | 92 | 86 | 98 |
| Wisconsin | 55 | 4154 | 4006448 | 104 | 91 | 88 | 94 |
| Connecticut | 9 | 2557 | 2380472 | 107 | 90 | 86 | 93 |
| Massachusetts | 25 | 4821 | 4583468 | 105 | 90 | 87 | 92 |
| Alaska | 2 | 190 | 339256 | 56 | 82 | 70 | 94 |
| Oregon | 41 | 2388 | 2600424 | 92 | 82 | 78 | 85 |
| Colorado | 8 | 2214 | 2858791 | 77 | 81 | 77 | 84 |
| Montana | 30 | 644 | 705675 | 91 | 81 | 75 | 87 |
| Nebraska | 31 | 1046 | 1208483 | 87 | 72 | 68 | 77 |
| Hawaii | 15 | 184 | 264081 | 70 | 69 | 59 | 79 |
| Minnesota | 27 | 2451 | 3654613 | 67 | 62 | 60 | 65 |
| Utah | 49 | 856 | 1353292 | 63 | 62 | 58 | 66 |

Table S3. Age-adjusted coronary heart disease mortality rate per 100,000 by state in non-Hispanic European American men aged 35-84 years: United States, 2005-2007

| State | State Code | Deaths | Population | Crude Rate | Age Adjusted Rate | Age Adjusted Rate Lower 95% Confidence Interval | Age Adjusted Rate Upper 95% Confidence Interval |
| --- | --- | --- | --- | --- | --- | --- | --- |
| Oklahoma | 40 | 7408 | 2045716 | 362 | 346 | 338 | 354 |
| Tennessee | 47 | 11798 | 3721770 | 317 | 326 | 320 | 331 |
| West Virginia | 54 | 4456 | 1363248 | 327 | 316 | 306 | 325 |
| Arkansas | 5 | 5566 | 1688098 | 330 | 312 | 303 | 320 |
| Mississippi | 28 | 4134 | 1339916 | 309 | 301 | 291 | 310 |
| Kentucky | 21 | 8060 | 2830179 | 285 | 299 | 292 | 306 |
| Rhode Island | 44 | 2003 | 678510 | 295 | 292 | 280 | 305 |
| New York | 36 | 28203 | 9549055 | 295 | 290 | 287 | 293 |
| Missouri | 29 | 10463 | 3721030 | 281 | 283 | 278 | 289 |
| Ohio | 39 | 20273 | 7387531 | 274 | 282 | 278 | 286 |
| Louisiana | 22 | 5888 | 2107759 | 279 | 280 | 273 | 287 |
| Michigan | 26 | 16326 | 6252001 | 261 | 273 | 269 | 277 |
| Indiana | 18 | 10135 | 4020812 | 252 | 266 | 261 | 272 |
| Texas | 48 | 23321 | 9063615 | 257 | 265 | 262 | 268 |
| Iowa | 19 | 5657 | 2086835 | 271 | 264 | 257 | 271 |
| Delaware | 10 | 1335 | 480054 | 278 | 261 | 247 | 275 |
| Nevada | 32 | 3445 | 1302662 | 264 | 259 | 250 | 268 |
| California | 6 | 34806 | 13487404 | 258 | 257 | 254 | 259 |
| Pennsylvania | 42 | 22261 | 8258920 | 270 | 257 | 254 | 261 |
| North Carolina | 37 | 11985 | 4804592 | 249 | 256 | 251 | 260 |
| New Jersey | 34 | 11474 | 4526997 | 253 | 253 | 249 | 258 |
| Illinois | 17 | 16204 | 6620508 | 245 | 252 | 248 | 255 |
|  |  | 403345 | 1.6E+08 | 252 | 252 | 251 | 253 |
| South Carolina | 45 | 5825 | 2271374 | 256 | 251 | 245 | 258 |
| South Dakota | 46 | 1410 | 532878 | 265 | 251 | 238 | 264 |
| Maryland | 24 | 6313 | 2672589 | 236 | 247 | 241 | 253 |
| North Dakota | 38 | 1131 | 440254 | 257 | 245 | 231 | 259 |
| Florida | 12 | 28812 | 9625157 | 299 | 239 | 237 | 242 |
| Alabama | 1 | 6011 | 2515971 | 239 | 235 | 229 | 241 |
| Arizona | 4 | 8159 | 3077491 | 265 | 230 | 225 | 235 |
| Kansas | 20 | 3865 | 1711537 | 226 | 225 | 218 | 232 |
| Virginia | 51 | 8706 | 4126327 | 211 | 225 | 221 | 230 |
| Georgia | 13 | 8630 | 4311519 | 200 | 224 | 220 | 229 |
| Wyoming | 56 | 774 | 360249 | 215 | 224 | 208 | 240 |
| New Mexico | 35 | 1788 | 715204 | 250 | 222 | 212 | 233 |
| Washington | 53 | 8154 | 3976147 | 205 | 222 | 217 | 227 |
| Wisconsin | 55 | 8152 | 3846819 | 212 | 220 | 215 | 225 |
| New Hampshire | 33 | 1938 | 1000443 | 194 | 217 | 207 | 226 |
| Maine | 23 | 2225 | 1043219 | 213 | 216 | 207 | 226 |
| Vermont | 50 | 976 | 484924 | 201 | 216 | 202 | 230 |
| District of Columbia | 11 | 248 | 138753 | 179 | 215 | 188 | 242 |
| Idaho | 16 | 1952 | 932110 | 209 | 214 | 205 | 224 |
| Massachusetts | 25 | 8223 | 4141092 | 199 | 205 | 200 | 209 |
| Oregon | 41 | 4958 | 2442699 | 203 | 202 | 197 | 208 |
| Alaska | 2 | 506 | 368295 | 137 | 196 | 177 | 215 |
| Montana | 30 | 1382 | 686264 | 201 | 196 | 185 | 206 |
| Connecticut | 9 | 4059 | 2176919 | 186 | 189 | 183 | 195 |
| Colorado | 8 | 4345 | 2747204 | 158 | 184 | 178 | 189 |
| Hawaii | 15 | 496 | 293006 | 169 | 180 | 164 | 197 |
| Nebraska | 31 | 2051 | 1134694 | 181 | 179 | 171 | 187 |
| Minnesota | 27 | 5214 | 3500103 | 149 | 160 | 156 | 165 |
| Utah | 49 | 1841 | 1296839 | 142 | 153 | 146 | 160 |

SUPPLEMENTAL MATERIAL

(please see http://circulation.ahajournals.org)

Supplemental figures

Figure S1. Age-adjusted rate per 100,000 resident population of death from coronary heart disease by state for African American women aged 35-84 years: United States, 2005-2007. Legend: yellow 63-139, light orange >141-170, dark orange >173-188-130, red >199-259.


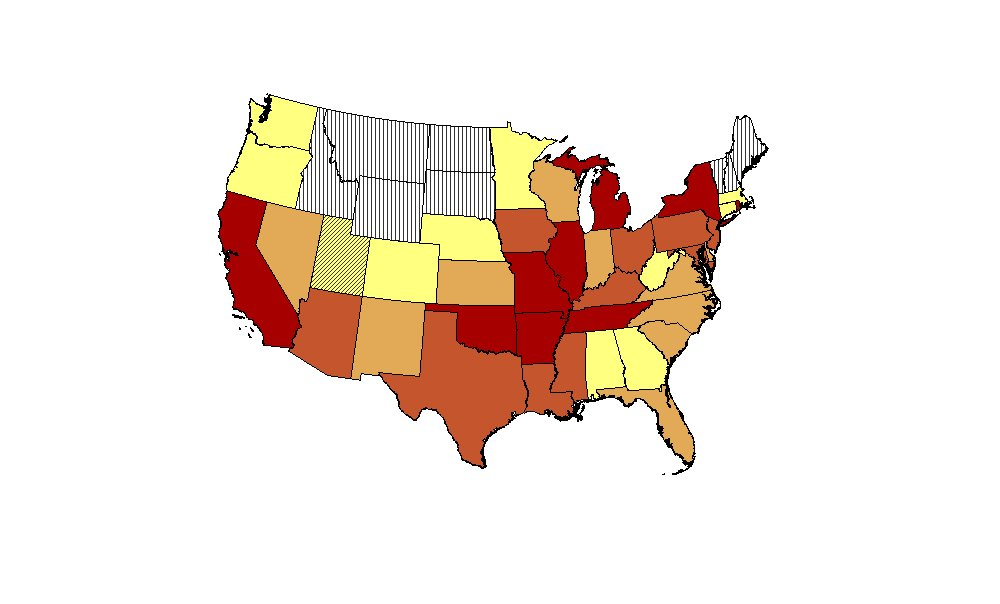


Figure S2. Age-adjusted rate of death per 100,000 population from coronary heart disease by state for African American men aged 35-84 years: United States, 2005-2007. Legend: yellow 88-238, light orange >243-304, dark orange >320-356, red >360-583.


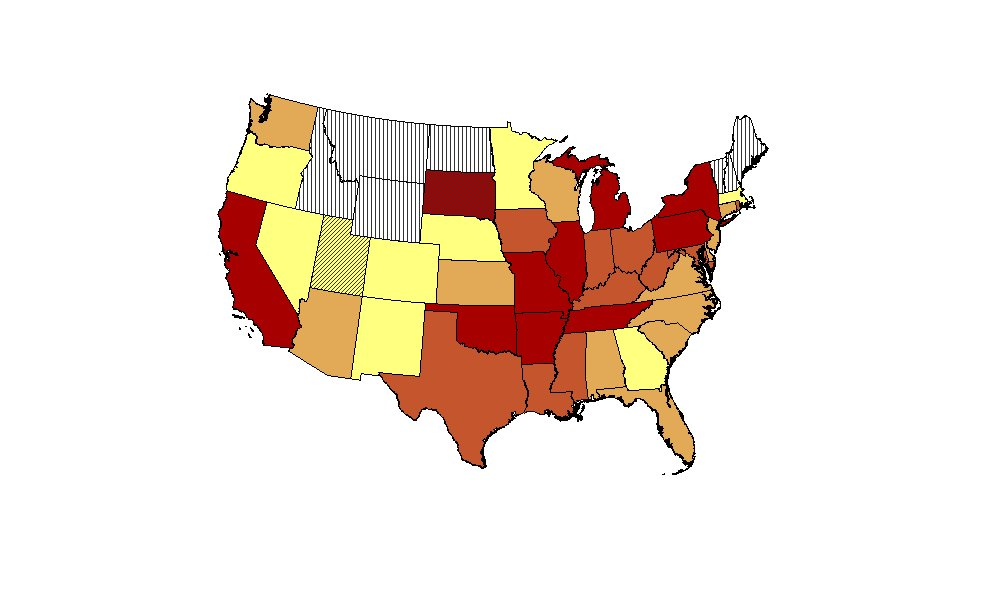

Supplement: Additional file 1 — Table S1. Classification rules used to assign counties to the six urbanization levels of the 2006 NCHS Urban-Rural Classification. [file 1471-2458-12-410-S1.doc]
