# Supplementary material for: Racial and geographic variation in coronary heart disease mortality trends
Source: BMC Public Health. 2012 Jun 6;12:410. doi: 10.1186/1471-2458-12-410 (PMC3532343; doi:10.1186/1471-2458-12-410)
Supplement: Additional file 2 — Table S2. Age-adjusted coronary heart disease mortality rate per 100,000 by state in non-Hispanic European American women aged 35-84 years: United States, 2005-2007. [file 1471-2458-12-410-S2.doc]

Table S2. Age-adjusted coronary heart disease mortality rate per 100,000 by state in non-Hispanic European American women aged 35-84 years: United States, 2005-2007

| State | State Code | Deaths | Population | Crude Rate | Age Adjusted Rate | Age Adjusted Rate Lower 95% Confidence Interval | Age Adjusted Rate Upper 95% Confidence Interval |
| --- | --- | --- | --- | --- | --- | --- | --- |
| Oklahoma | 40 | 4636 | 2227652 | 208 | 174 | 169 | 179 |
| Tennessee | 47 | 7170 | 4045305 | 177 | 158 | 154 | 162 |
| West Virginia | 54 | 2797 | 1479554 | 189 | 158 | 152 | 163 |
| New York | 36 | 19882 | 10400153 | 191 | 153 | 151 | 155 |
| Arkansas | 5 | 3282 | 1826844 | 180 | 150 | 144 | 155 |
| Kentucky | 21 | 4946 | 3097122 | 160 | 146 | 142 | 150 |
| Mississippi | 28 | 2459 | 1454669 | 169 | 142 | 136 | 147 |
| Rhode Island | 44 | 1341 | 758994 | 177 | 142 | 134 | 149 |
| Missouri | 29 | 6376 | 4016231 | 159 | 138 | 134 | 141 |
| Michigan | 26 | 10034 | 6607551 | 152 | 135 | 132 | 138 |
| Ohio | 39 | 12617 | 8034413 | 157 | 135 | 132 | 137 |
| Maryland | 24 | 4207 | 2894335 | 145 | 130 | 126 | 134 |
| Texas | 48 | 13823 | 9637353 | 143 | 129 | 127 | 131 |
| Louisiana | 22 | 3325 | 2271297 | 146 | 128 | 123 | 132 |
| Indiana | 18 | 6118 | 4327793 | 141 | 126 | 123 | 129 |
| California | 6 | 21141 | 14084624 | 150 | 125 | 124 | 127 |
| New Jersey | 34 | 7567 | 4960576 | 153 | 123 | 120 | 126 |
| Iowa | 19 | 3351 | 2224728 | 151 | 122 | 118 | 126 |
| Delaware | 10 | 765 | 526440 | 145 | 121 | 112 | 130 |
| Pennsylvania | 42 | 14111 | 9033598 | 156 | 121 | 119 | 123 |
|  |  | 239422 | 171371666 | 140 | 119 | 119 | 120 |
| Alabama | 1 | 3807 | 2733028 | 139 | 118 | 114 | 121 |
| Nevada | 32 | 1613 | 1274017 | 127 | 116 | 111 | 122 |
| Illinois | 17 | 9620 | 7135657 | 135 | 115 | 112 | 117 |
| New Mexico | 35 | 1042 | 777772 | 134 | 110 | 103 | 116 |
| North Carolina | 37 | 6515 | 5246953 | 124 | 110 | 108 | 113 |
| Arizona | 4 | 4592 | 3293390 | 139 | 109 | 106 | 112 |
| Florida | 12 | 16277 | 10387342 | 157 | 109 | 107 | 111 |
| South Carolina | 45 | 3047 | 2462076 | 124 | 107 | 104 | 111 |
| Wyoming | 56 | 405 | 362919 | 112 | 107 | 97 | 118 |
| South Dakota | 46 | 705 | 551973 | 128 | 104 | 96 | 111 |
| Vermont | 50 | 569 | 518138 | 110 | 104 | 96 | 113 |
| District of Columbia | 11 | 125 | 126593 | 99 | 103 | 85 | 122 |
| New Hampshire | 33 | 1082 | 1056316 | 102 | 101 | 95 | 107 |
| Virginia | 51 | 4818 | 4435667 | 109 | 101 | 98 | 104 |
| Washington | 53 | 4393 | 4154917 | 106 | 101 | 98 | 104 |
| Kansas | 20 | 2095 | 1835498 | 114 | 98 | 93 | 102 |
| Georgia | 13 | 4569 | 4607765 | 99 | 96 | 94 | 99 |
| Maine | 23 | 1205 | 1127885 | 107 | 95 | 90 | 101 |
| North Dakota | 38 | 545 | 455402 | 120 | 95 | 87 | 103 |
| Idaho | 16 | 945 | 964123 | 98 | 92 | 86 | 98 |
| Wisconsin | 55 | 4154 | 4006448 | 104 | 91 | 88 | 94 |
| Connecticut | 9 | 2557 | 2380472 | 107 | 90 | 86 | 93 |
| Massachusetts | 25 | 4821 | 4583468 | 105 | 90 | 87 | 92 |
| Alaska | 2 | 190 | 339256 | 56 | 82 | 70 | 94 |
| Oregon | 41 | 2388 | 2600424 | 92 | 82 | 78 | 85 |
| Colorado | 8 | 2214 | 2858791 | 77 | 81 | 77 | 84 |
| Montana | 30 | 644 | 705675 | 91 | 81 | 75 | 87 |
| Nebraska | 31 | 1046 | 1208483 | 87 | 72 | 68 | 77 |
| Hawaii | 15 | 184 | 264081 | 70 | 69 | 59 | 79 |
| Minnesota | 27 | 2451 | 3654613 | 67 | 62 | 60 | 65 |
| Utah | 49 | 856 | 1353292 | 63 | 62 | 58 | 66 |
