# Supplementary material for: Racial and geographic variation in coronary heart disease mortality trends
Source: BMC Public Health. 2012 Jun 6;12:410. doi: 10.1186/1471-2458-12-410 (PMC3532343; doi:10.1186/1471-2458-12-410)
Supplement: Additional file 3 — Table S3. Age-adjusted coronary heart disease mortality rate per 100,000 by state in non-Hispanic European American men aged 35-84 years: United States, 2005-2007. [file 1471-2458-12-410-S3.doc]

Table S3. Age-adjusted coronary heart disease mortality rate per 100,000 by state in non-Hispanic European American men aged 35-84 years: United States, 2005-2007

| State | State Code | Deaths | Population | Crude Rate | Age Adjusted Rate | Age Adjusted Rate Lower 95% Confidence Interval | Age Adjusted Rate Upper 95% Confidence Interval |
| --- | --- | --- | --- | --- | --- | --- | --- |
| Oklahoma | 40 | 7408 | 2045716 | 362 | 346 | 338 | 354 |
| Tennessee | 47 | 11798 | 3721770 | 317 | 326 | 320 | 331 |
| West Virginia | 54 | 4456 | 1363248 | 327 | 316 | 306 | 325 |
| Arkansas | 5 | 5566 | 1688098 | 330 | 312 | 303 | 320 |
| Mississippi | 28 | 4134 | 1339916 | 309 | 301 | 291 | 310 |
| Kentucky | 21 | 8060 | 2830179 | 285 | 299 | 292 | 306 |
| Rhode Island | 44 | 2003 | 678510 | 295 | 292 | 280 | 305 |
| New York | 36 | 28203 | 9549055 | 295 | 290 | 287 | 293 |
| Missouri | 29 | 10463 | 3721030 | 281 | 283 | 278 | 289 |
| Ohio | 39 | 20273 | 7387531 | 274 | 282 | 278 | 286 |
| Louisiana | 22 | 5888 | 2107759 | 279 | 280 | 273 | 287 |
| Michigan | 26 | 16326 | 6252001 | 261 | 273 | 269 | 277 |
| Indiana | 18 | 10135 | 4020812 | 252 | 266 | 261 | 272 |
| Texas | 48 | 23321 | 9063615 | 257 | 265 | 262 | 268 |
| Iowa | 19 | 5657 | 2086835 | 271 | 264 | 257 | 271 |
| Delaware | 10 | 1335 | 480054 | 278 | 261 | 247 | 275 |
| Nevada | 32 | 3445 | 1302662 | 264 | 259 | 250 | 268 |
| California | 6 | 34806 | 13487404 | 258 | 257 | 254 | 259 |
| Pennsylvania | 42 | 22261 | 8258920 | 270 | 257 | 254 | 261 |
| North Carolina | 37 | 11985 | 4804592 | 249 | 256 | 251 | 260 |
| New Jersey | 34 | 11474 | 4526997 | 253 | 253 | 249 | 258 |
| Illinois | 17 | 16204 | 6620508 | 245 | 252 | 248 | 255 |
|  |  | 403345 | 1.6E+08 | 252 | 252 | 251 | 253 |
| South Carolina | 45 | 5825 | 2271374 | 256 | 251 | 245 | 258 |
| South Dakota | 46 | 1410 | 532878 | 265 | 251 | 238 | 264 |
| Maryland | 24 | 6313 | 2672589 | 236 | 247 | 241 | 253 |
| North Dakota | 38 | 1131 | 440254 | 257 | 245 | 231 | 259 |
| Florida | 12 | 28812 | 9625157 | 299 | 239 | 237 | 242 |
| Alabama | 1 | 6011 | 2515971 | 239 | 235 | 229 | 241 |
| Arizona | 4 | 8159 | 3077491 | 265 | 230 | 225 | 235 |
| Kansas | 20 | 3865 | 1711537 | 226 | 225 | 218 | 232 |
| Virginia | 51 | 8706 | 4126327 | 211 | 225 | 221 | 230 |
| Georgia | 13 | 8630 | 4311519 | 200 | 224 | 220 | 229 |
| Wyoming | 56 | 774 | 360249 | 215 | 224 | 208 | 240 |
| New Mexico | 35 | 1788 | 715204 | 250 | 222 | 212 | 233 |
| Washington | 53 | 8154 | 3976147 | 205 | 222 | 217 | 227 |
| Wisconsin | 55 | 8152 | 3846819 | 212 | 220 | 215 | 225 |
| New Hampshire | 33 | 1938 | 1000443 | 194 | 217 | 207 | 226 |
| Maine | 23 | 2225 | 1043219 | 213 | 216 | 207 | 226 |
| Vermont | 50 | 976 | 484924 | 201 | 216 | 202 | 230 |
| District of Columbia | 11 | 248 | 138753 | 179 | 215 | 188 | 242 |
| Idaho | 16 | 1952 | 932110 | 209 | 214 | 205 | 224 |
| Massachusetts | 25 | 8223 | 4141092 | 199 | 205 | 200 | 209 |
| Oregon | 41 | 4958 | 2442699 | 203 | 202 | 197 | 208 |
| Alaska | 2 | 506 | 368295 | 137 | 196 | 177 | 215 |
| Montana | 30 | 1382 | 686264 | 201 | 196 | 185 | 206 |
| Connecticut | 9 | 4059 | 2176919 | 186 | 189 | 183 | 195 |
| Colorado | 8 | 4345 | 2747204 | 158 | 184 | 178 | 189 |
| Hawaii | 15 | 496 | 293006 | 169 | 180 | 164 | 197 |
| Nebraska | 31 | 2051 | 1134694 | 181 | 179 | 171 | 187 |
| Minnesota | 27 | 5214 | 3500103 | 149 | 160 | 156 | 165 |
| Utah | 49 | 1841 | 1296839 | 142 | 153 | 146 | 160 |
