# Supplementary material for: Racial and geographic variation in coronary heart disease mortality trends
Source: BMC Public Health. 2012 Jun 6;12:410. doi: 10.1186/1471-2458-12-410 (PMC3532343; doi:10.1186/1471-2458-12-410)

SUPPLEMENTAL MATERIAL

(please see http://circulation.ahajournals.org)

Supplemental figures

Figure S1. Age-adjusted rate per 100,000 resident population of death from coronary heart disease by state for African American women aged 35-84 years: United States, 2005-2007. Legend: yellow 63-139, light orange >141-170, dark orange >173-188-130, red >199-259.


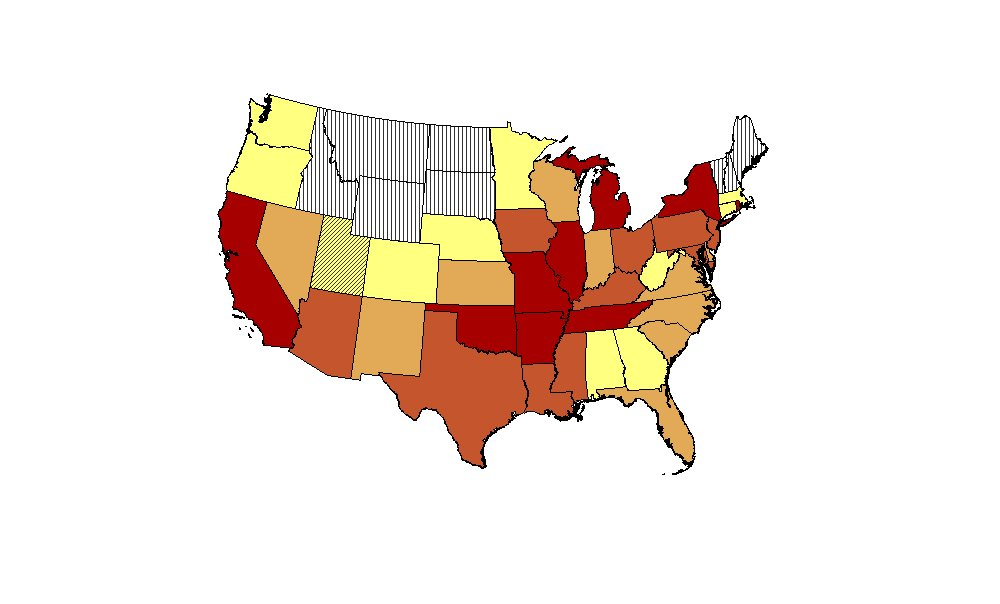

Supplement: Additional file 4 — Figure S1. Age-adjusted rate per 100,000 resident population of death from coronary heart disease by state for African American women aged 35–84 years: United States, 2005–2007. Legend: yellow 63-139, light orange >141-170, dark orange >173-188, red >199-259. [file 1471-2458-12-410-S4.doc]
