# Supplementary material for: Racial and geographic variation in coronary heart disease mortality trends
Source: BMC Public Health. 2012 Jun 6;12:410. doi: 10.1186/1471-2458-12-410 (PMC3532343; doi:10.1186/1471-2458-12-410)

Figure S2. Age-adjusted rate of death per 100,000 population from coronary heart disease by state for African American men aged 35-84 years: United States, 2005-2007. Legend: yellow 88-238, light orange >243-304, dark orange >320-356, red >360-583.


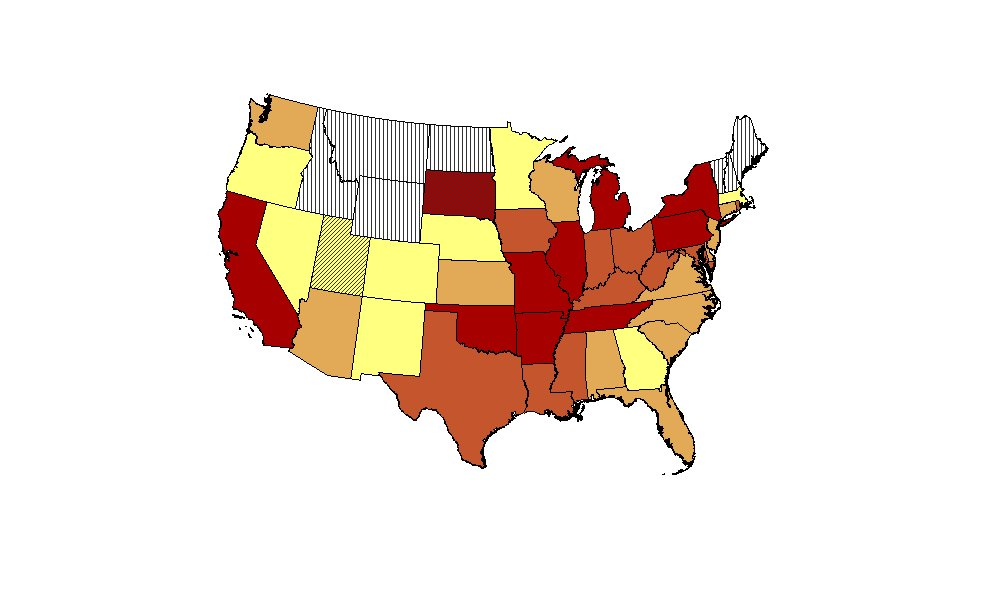

Supplement: Additional file 5 — Figure S2. Age-adjusted rate of death per 100,000 population from coronary heart disease by state for African American men aged 35–84 years: United States, 2005–2007. Legend: yellow 88–238, light orange >243-304, dark orange >320-356, red >360-583. [file 1471-2458-12-410-S5.doc]
